# Effect of DanQi Pill on PPARα, lipid disorders and arachidonic acid pathway in rat model of coronary heart disease

**DOI:** 10.1186/s12906-016-1083-3

**Published:** 2016-03-22

**Authors:** Hong Chang, Qiyan Wang, Tianjiao Shi, Kuiyuan Huo, Chun Li, Qian Zhang, Guoli Wang, Yuanyuan Wang, Binghua Tang, Wei Wang, Yong Wang

**Affiliations:** Beijing University of Chinese Medicine, Beijing, 100029 China; Modern Research Center for Traditional Chinese Medicine, Beijing University of Chinese Medicine, Beijing, 100029 China; Tangshan People’s hospital, Tangshan, Hebei 063001 China

**Keywords:** Chinese herbs, Lipid disorders, Arachidonic acid, Coronary heart disease

## Abstract

**Background:**

Danqi pill (DQP) is one of the most widely prescribed formulas and has been shown to have remarkable protective effect on coronary heart disease (CHD). However, its regulatory effects on lipid metabolism disorders haven’t been comprehensively studied so far. We aimed to explore the effects of DQP on Peroxisome Proliferator activated receptors α (PPARα), lipid uptake-transportation-metabolism pathway and arachidonic acid (AA)-mediated inflammation pathway in rats with CHD.

**Methods:**

80 Sprague-Dawley (SD) Rats were randomly divided into sham group, model group, positive control group and DQP group. Rat model of CHD was induced by ligation of left ventricle anterior descending artery and fed with high fat diet in all but the sham group. Rats in sham group only underwent thoracotomy. After surgery, rats in the positive control and DQP group received daily treatments of pravastatin and DQP respectively. At 28 days after surgery, rats were sacrificed and plasma lipids were evaluated by plasma biochemical detection. Western blot and PCR were applied to evaluate the expressions of PPARα, proteins involved in lipid metabolism and AA pathways.

**Results:**

Twenty eight days after surgery, dyslipidemia developed in CHD model rats, as illustrated by elevated plasma lipid levels. Expressions of apolipoprotein A-I (ApoA-I), cluster of differentiation 36 (CD36) and fatty acid binding protein (FABP) in the heart tissues of model group were down-regulated compared with those in sham group. Expressions of carnitine palmitoyl transferase I (CPT-1A) and lipoproteinlipase (LPL) were also reduced significantly. In addition, levels of phospholipase A2 (PLA2) and cyclooxygenase 2 (COX-2) were up-regulated. Expressions of Nuclear factor-κB (NF- κB) and signal transducer and activator of transcription 3 (STAT3) also increased. Furthermore, Expression of PPARα decreased in the model group. DQP significantly up-regulated expressions of ApoA-I and FABP, as well as the expressions of CPT-1A and CD36. In addition, DQP down-regulated expressions of PLA2, COX-2 and NF-κB in inflammation pathway. Levels of STAT3 and LPL were not affected by DQP treatment. In particular, DQP up-regulated PPARα level significantly.

**Conclusions:**

DQP could effectively regulate lipid uptake-transportation-metabolism process in CHD model rats, and the effect is achieved mainly by activating ApoA-I-CD36-CPT-1A molecules. Interestingly, DQP can up-regulate expression of PPARα significantly. The anti-inflammatory effect of DQP is partly exerted by inhibiting expressions of PLA2-COX2 -NF-κB pathway.

## Background

Coronary heart disease (CHD) remains one of the major causes of mortality worldwide, even though great progress has been made in the development of novel therapeutic approaches. Pathological mechanisms of CHD have been the focus of research and it’s believed that lipid infiltration and inflammation contribute to CHD progress [1, 2]. The corresponding anti-inflammatory and lipid-lowering drugs have been widely applied in the management of CHD [3–5].

According to lipid infiltration theory of CHD, abnormal deposition of lipid in subendothelial layer of artery causes narrowing of coronary arteries, which will eventually lead to CHD [6]. Studies have shown that high level of plasma low density lipoprotein (LDL) is an independent risk factor for CHD, whereas high level of high density lipoprotein (HDL) is inversely correlated with risk of CHD [7, 8]. Apolipoprotein A-I (ApoA-I) is the major protein component of HDL and is involved in the transport of cholesterol from circulatory system to the liver [9]. Lipoprotein lipase (LPL) is the key enzyme in the catabolism of triglyceride. It breaks down triglycerides into free fatty acids (FFA), which provide 80 % of the energy consumed by cardiomyocytes [10]. Plasma FFA are combined with fatty acid-binding protein (FABP) and transferred into cytoplasm of cardiac cells by cluster of differentiation 36 (CD36), and then further transported into mitochondria through carnitine, catalyzed by carnitinepalmitoyltransferase I (CPT-I) which is another key enzyme in fatty acid metabolism [11, 12]. FFA eventually undergo oxidation in mitochondria to produce ATP for heart contraction [13, 14].

In addition to lipid filtration, inflammation also contributes to the initiation and progression of CHD. Arachidonic acid (AA) mediated inflammation is thought to be the major cause of myocardial cell damage [15]. Under inflammatory stimuli, phospholipase A2 (PLA2) hydrolyzes phospholipids in cell membranes and releases AA, which are further converted into active compounds catalyzed by different subtypes of cyclooxygenases (COXs). The major metabolic products of AA include prostaglandins (PGs), prostacyclin (PGI2) and thromboxanes (TXBs), which are involved in inflammatory responses [16, 17]. Therefore, inhibiting AA-mediated PLA2-COX pathway to protect cardiac cells against inflammatory injuries has become the strategy for the management of CHD [18]. Nonsteroidal anti-inflammatory drugs (NSAIDs) have been widely used to treat patients with CHD. However, there has been an increasing number of reports about cardiotoxicities induced by NSAIDs [19].

Peroxisome Proliferator activated receptors α (PPARα) has been found to play crucial roles in regulating lipid metabolism [20, 21]. Activation of PPARα reduces plasma level of very low density lipoprotein (VLDL) by inhibiting synthesis of triglyceride and cholesterol. PPARα also promotes plasma triglyceride clearance by mobilizing LPL activity and enhancing oxidation of fatty acids, thereby regulating plasma lipid levels [22]. In addition, PPARα also exerts anti-inflammatory effect, mainly through inhibiting the activity of COX-2 and monocyte chemoattractant protein-1 (MCP-1) through NF-κB signaling pathway [23, 24]. Therefore, PPARα agonists are considered to be the promising new candidates for the treatment of CHD.

Traditional Chinese medicine has been applied in the treatment of CHD for thousands of years. DQP is one of the most widely prescribed formulas and has been shown to have remarkable protective effect on ischemia heart tissues [25]. DQP is prepared from equal amounts of two dried Chinese herbs, *Salvia miltiorrhiza Bunge* and *Panax notoginseng (Burk.) F. H. Chen*. The major effective ingredients of *Salvia miltiorrhiza Bunge* and *Panax notoginseng (Burk.) F. H. Chen* are salvianolic acids and *panax notoginseng saponins* (PNS), respectively [26, 27]. DQP was widely produced in China in accordance with the China Pharmacopoeia standard of quality control, and was listed in Chinese Pharmacopoeia 2010 as routine drug in the clinical treatment of CHD [28]. Until now, many studies have been conducted to investigate the effects of active monomers in DQP on CHD. PNS were found to inhibit ischemia-induced myocardial apoptosis and tanshinone IIA (monomer of *Salvia miltiorrhiza*) was found to have cardioprotective and anti-hypertrophic effects [29, 30]. Our previous studies proved that DQP had efficacy on CHD through regulating Renin-Angiotensin-Aldosterone System (RAAS) and lipid metabolism [31, 32]. However, the comprehensive effects of DQP on PPARα, lipid metabolism and inflammation pathway mediated by PLA2-COX-2 haven’t been completely understood yet. In this study, we aim to investigate the effects of DQP on lipid metabolism and PLA2-COX-2 pathway, so as to uncover the pharmacological mechanisms of DQP and provide experimental basis for its clinical application.

## Methods

### Animals and grouping

Studies were performed in accordance with the Guide for the Care and Use of Laboratory Animals published by the National Institutes of Health (NIH Publications No. 85-23, revised 1996) the China Physiological Society’s “Guiding Principles in the Care and Use of Animals” and with approval of the Animal Care Committee of Beijing Medical Center. A total of 80 male Sprague-Dawley (SD) rats (weighted 220 g ± 10 g) in Specific Pathogen Free (SPF) grade were selected (purchased from Beijing Vital River Laboratory Animal Technology Co.Ltd. License number: SCXK2010–2011).

### Animal model preparation

Rats were randomly divided into four groups: sham group, model group, positive control group (control) and DQP group. CHD rat model with in vivo coronary left anterior descending (LAD) occlusion was then induced as described in our previous study [32]. Briefly, left thoracotomy was performed after rats were anaesthetized by 1 % pentobarbital sodium at the dosage of 50 mg/kg. LAD coronary artery was then ligated with a 5-0 polypropylene suture. The thorax was closed with 2.0 absorbable silk surgical sutures as we previously reported after ligation. The sodium penicillin was given to prevent the potential wound inflammation for 3 days after surgery. Rats in sham group received the same procedures except that the coronary artery was not ligated. In addition, rats in model group, positive control group and DQP group were fed with high fat diet for 28 days while rats in sham group fed with normal diet. From the second day after operation, rats in DQP group were treated with DQP aqueous solution (Tongren tang, Beijing, China, Series: 6128006) at the daily dosage of 1.5 mg/kg via oral gavage for 28 consecutive days. Rats in positive control group received pravastatin (Bristol-Myers Squibb, China, Series: H19980197) aqueous solution at the daily dosage of 1.2 mg/kg. Animals in sham group and model group were given a gavage of normal saline water for 28 days. All animals were anaesthetized using pentobarbital sodium following an overnight fast at the end of study. Blood samples were collected via abdominal aorta puncture.

The overall mortality rate of rats during the entire experiment (28 days after surgery) was 22.5 %. Among them, 7 rats died during the surgery and 11 rats died the day after the surgery, probably due to acute pump failure or lethal arrhythmias, as they manifested serious clinical signs, such as short of breath and severe tachycardia.

### Assessment of cardiac functions

Cardiac functions were assessed by echocardiography at 28d after surgery. Rats were anaesthetized before assessment. The parameters of heart functions include: left ventricular end-systolic diameter (LVESd), left ventricular end-diastolic diameter (LVEDd), ejection fraction (EF) and fractional shortening (FS). EF was calculated as follows: EF = [(LVEDv − LVESv)/LVEDv] × 100 %. FS was calculated using the equation: [(LVEDd − LVESd)/LVEDd] × 100 %.

### Measurement of plasma TC, TG, LDL and HDL levels

Twenty eight days after surgery, animals were sacrificed after anaesthetization and blood was taken through abdominal aorta. The blood was centrifuged at 8000 g for 10 min and supernatant was used for detection of plasma indicators. Plasma levels of total cholesterol (TC), triglyceride (TG), high density lipoprotein (HDL) and low density lipoprotein (LDL) were measured by automatic biochemical analyzer (HITACH17080, Japan) following manufacturer’s instructions. The cholesterol kit (COD-PAP Method), triglyceride kit (GPO-PAP Method), direct HDL kit and direct LDL kit were purchased from BiosinaTechnologies Inc.

### Evaluation of mRNA expressions of molecules involved in lipid metabolism by polymerase chain reaction (PCR)

Expressions of LPL and CD36 in heart tissues were determined by reverse transcriptase polymerase chain reaction (RT-PCR). Heart tissues near infarct zone were excised and frozen in liquid nitrogen. Total RNA was extracted using Trizol Reagent (Gibco-BRL, Paisley, UK). The concentration of RNA was measured by Nano Drop 2000 (Thermo Scientific, USA). RNA was transcribed using the RevertAidTM First Stand cDNASynthesis kit (Fermentas, LT). RNAs quantities of LPL, CD36 and GAPDH were analyzed by PCR. The reaction system contained 2 μl cDNA, 0.5 μl forward and reverse primers, 10 μl Rox and 7 μl DEPC water. PCR conditions were set as follows: 15 s at 95 °C for denaturation, 1 min at 55 °C for annealing and extension. 40 cycles of amplifications were performed for each gene. The sequences of the primers were shown at Table 1. Quantities of LPL and CD36 mRNA were normalized to mRNA quantities of GAPDH.

**Table 1:** Nucleotide sequences of primers used in real-time PCR

Gene (accession no.)	Primes	Nucleotiede sequences5‘-3’	Length (bp)	Temp (°C)
LPL	Forward	CGCTCCATCCATCTCTTC		57.3
	Reverse	GGCTCTGACCTTGTTGAT	159	55.0
CD36	Forward	GGTCCTTACACATACAGAGT		55.8
	Reverse	CCACAGCCAGATTGAGAA	163	55.0
GAPDH	Forward	TCAACGGCACAGTCAAG		55.0
	Reverse	TACTCAGCACCAGCATCA	116	55.0

### Measurement of protein expressions detected by western blot

The border of infarcted myocardium in left ventricle homogenates was prepared for the analysis of protein levels. Equal amounts of protein extracts (20 μg) were separated by 12.5 % or 15 % sodium dodecyl sulphate (SDS)-polyacrylamide gel electrophoresis (Bio-Rad, CA, U.S.A.) and transferred to nitrocellulose membranes electrophoretically (semidry transfer). Membranes were blocked with 5 % non-fat dry milk in Tris-buffered saline (20 mM Tris, pH 7.6, 137 mM NaCl) with 0.1 % Tween 20, washed, and then incubated with primary antibody. Primary antibodies employed included: goat polyclonal antiglyceraldehyde-3-phosphate dehydrogenase (GAPDH) and anti-ApoA-I, PPARα, FABP, CPT-1A, PLA2, COX-2, NF-κB and STAT3 (Anti-Apolipoprotein AIantibody, ab334707, 1:250; Anti-CPT1A,ab128568,1:500;Anti-PPARα antibody,ab8934,1:1000;Anti-STAT3 antibody, ab76315,1:250; Anti-Cardiac FABP antibody, ab174673,1:2000;Anti-Phospholipase A2 Antibody, ab23705, 1:250;Anti-NF-κB p105/p50 antibody,ab32360,1:500; Anti-COX2, ab15191,1:500). The primary antibody was firstly incubated, and then the secondary antibodies (Santa Cruz Biotechnology Inc., CA, U.S.A.) was added. After exposed by chemiluminescence developing agents, the protein levels and GAPDH in each sample were evaluated. The gel was scanned and the band densities were quantified. Protein expressions were normalized by the GAPDH band densities and then normalized by density in sham group to determine their concentrations.

### Statistical analysis

Data were presented as mean ± standard deviation (mean ± SD). Differences among groups were analyzed by one-way analysis of variance (ANOVA) test. *P* values of smaller than 0.05 were considered as statistically significant. All statistical analyses were carried out using SPSS 17.0 software.

## Results

### Effects of DQP on cardiac functions

**Fig. 1:**
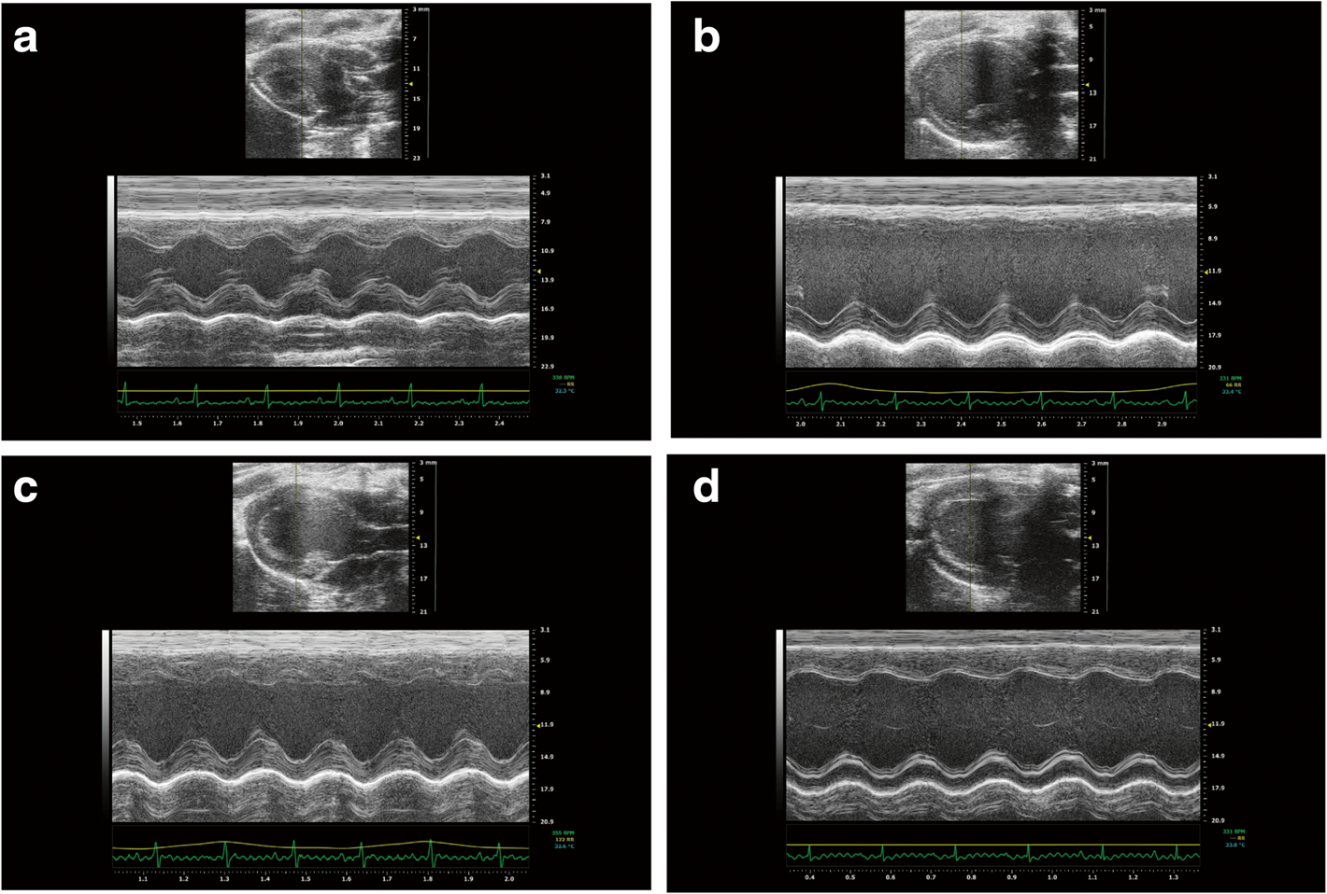
DQP improved parameters of cardiac functions detected by echocardiography. Echocardiography results showed that LVEDd and LVESd in the model group increased significantly compared with those in the sham group. EF and FS in model group decreased respectively. DQP could reduce the LVESd while LVEDd wasn’t affected by DQP. DQP also could upregulate EF and FS respectively, suggesting that DQP could improve cardiac function (*P < 0.05, **P < 0.01, other groups vs. model group, n = 8). **a** Cardiac functions in sham-operated group. **b** Cardiac functions were down-regulated in model group rats. **c** Positive Drug had no effects on the cardiac function. **d** DQP could significantly up-regulate the EF and FS

Echocardiography results showed that LVEDd and LVESd in the model group increased by 53.52 % (*P* < 0.01) and 156.73 % (*P* < 0.01) as compared with those in the sham group, respectively (Fig. 1, Table 2). EF and FS in model group decreased by 49.96 % (*P < 0.001*) and 58.30 % (*P < 0.001*) respectively, suggesting that cardiac functions were severely impaired and CHD model was successfully induced. After treatment with DQP for 28 days, LVESd was reduced as compared with that in the model group. LVEDd wasn’t affected by DQP. DQP could elevate the values of EF and FS by 43.00 % (*P* = 0.036) and 54.06 % (*P* = 0.041), respectively.

**Table 2:** Indicators of cardiac function in different groups

Group	Sham	Model	Control	DQP
LVEDd(mm)	5.651 ± 0.849**	8.675 ± 1.335	9.043 ± 1.254	8.812 ± 0.557
LVEDs(mm)	2.755 ± 0.848**	7.073 ± 1.716	6.635 ± 0.769*	6.748 ± 0.677
EF(%)	81.604 ± 7.922**	40.831 ± 19.212	32.327 ± 16.765	58.391 ± 10.623*
FS(%)	51.939 ± 9.322**	21.661 ± 11.782	16.579 ± 9.468	33.372 ± 6.696*

Compared with model group, *P* < 0.05 *, *P* < 0.01**

### Effects of DQP on plasma lipid and lipoprotein levels

Compared with the sham group, levels of TC, TG, LDL and VLDL in the model group increased by 40.50 % (*P* = 0.032), 155.33 % (*P* = 0.004), 49.07 % (*P* = 0.031) and 170.52 % (*P* = 0.002), respectively. HDL level decreased by 46.11 % (*P* = 0.021) in model group compared with that in the sham group. After treatment with DQP, TG level rather than TC level in DQP group was reduced in DQP group compared with model group, suggesting that DQP has better efficacy on TG rather than TC. Levels of LDL and VLDL in the DQP group decreased by31.85 % (*P* = 0.015) and 56.49 % (*P* = 0.008) respectively, as compared with those in the model group. HDL level was up-regulated by 76.44 % (*P* = 0.026). These data suggested that DQP could regulate disorders of lipid metabolism in rats with CHD. The effects of Pravastatin on plasma lipid levels were similar with those of DQP (Table 3).

**Table 3:** Indicators of plasma lipid and lipoprotein levels in different groups

Group	Sham	Model	Control	DQP
TC(mmol/L)	1.49 ± 0.35*	1.49 ± 0.35*	1.49 ± 0.35*	2.24 ± 0.28
TG(mmol/L)	1.05 ± 0.21**	2.68 ± 0.49	0.98 ± 0.51**	1.35 ± 0.37*
HDL(mmol/L)	0.83 ± 0.18*	0.44 ± 0.26	0.97 ± 0.20*	0.78 ± 0.35*
LDL(mmol/L)	0.50 ± 0.13*	0.75 ± 0.20	0.33 ± 0.16**	0.51 ± 0.11*
VLDL(mmol/L)	0.46 ± 0.104**	1.25 ± 0.238	0.45 ± 0.176**	0.54 ± 0.207**

Compared with model group, *P* < 0.05 *, *P* < 0.01**

### Effects of DQP on key molecules involved in uptake of lipids

Expressions of LPL and CD36 were down-regulated by 30.65 % (*P* = 0.002) and 9.2 % (*P* = 0.034) in model group compared with those in the sham group, which suggested that uptake of FFA by cardiocytes was compromised in the model rats. After treatment with DQP, expressions of CD36 was up-regulated by 15.47 % (*P* = 0.03), compared with those in the model group (Table 4). However, DQP showed no effect on expression of LPL in this study. Pravastatin showed no effect on the expressions of either LPL or CD36 (Table 4).

**Table 4:** Key molecules involved in uptake of lipid in different groups

Group	Sham	Model	Control	DQP
CD36	1.208 ± 0. 026*	1.097 ± 0.068	1.235 ± 0.149	1.267 ± 0.063*
LPL	1.525 ± 0. 159**	1.194 ± 0.247	0.893 ± 0.226	1.158 ± 0.0247

Compared with model group, *P* < 0.05 *, *P* < 0.01**

### Effects of DQP on lipid transportations

**Fig. 2:**
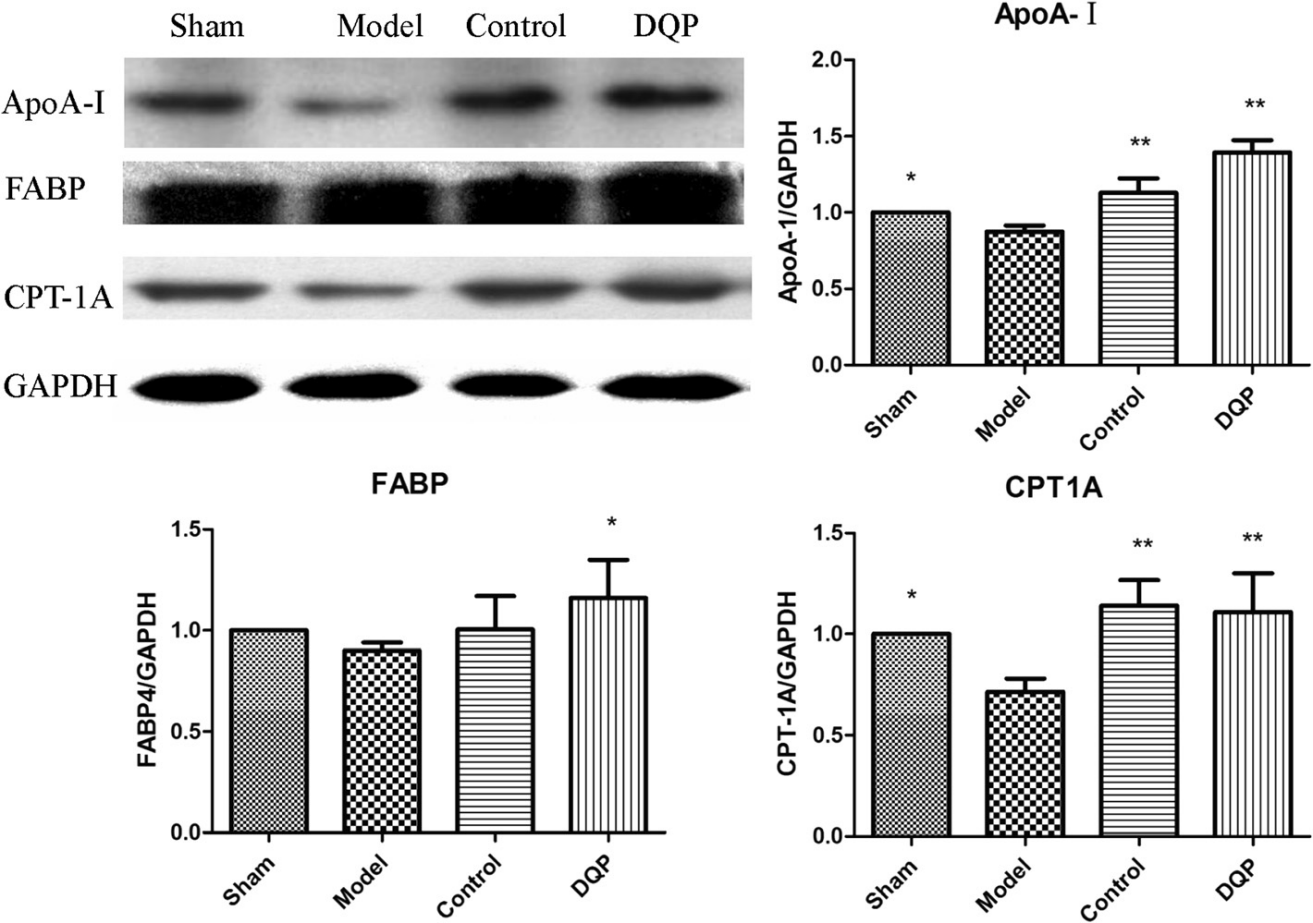
Effects of DQP on the myocardial concentrations of ApoA-I, FABP and CPT-1A. The results showed that expressions of ApoA-I and CPT-1A in the model group decreased compared with those in the sham group. Level of FABP showed no statistical difference between sham and model group. DQP could increase ApoA-I, FABP and CPT-1A levels. Pravastatin had similar effect as DQP. *indicates *P* < 0.05, **indicates *P* < 0.01. Levels in the model group (n = 6) were used as reference to calculate *P* values

Expressions of ApoA-I and FABP in the model group (0.87 ± 0.04 and 0.90 ± 0.039) were down-regulated by 12.63 % (*P* = 0.016) and 10.00 % (*P* = 0.381), respectively, as compared with those in the sham group (1.00 ± 0.00, 1.00 ± 0.00). Level of CPT-1A, the key enzyme in lipid oxidation, decreased by 28.44 % (0.71 ± 0.064, *P* = 0.017) in the model group compared with those in the sham group (1.00 ± 0.00). Suppressed expressions of ApoA-I, FABP and CPT-1A suggested that transportation of fatty acids into mitochondria for oxidation was inhibited in CHD rats. In DQP group, expressions of ApoA-I (1.39 ± 0.081) and FABP (1.16 ± 0.189) were up-regulated by 59.53 % (*P* < 0.01) and 28.97 % (*P* = 0.03) respectively, compared with those in the model group. Expression of CPT-1A also increased by 54.93 % (1.11 ± 0.192, *P* = 0.001), suggesting that DQP promoted the transportation of lipid into mitochondria for oxidation (Fig. 2).

### Effects of DQP on inflammation pathway

**Fig. 3:**
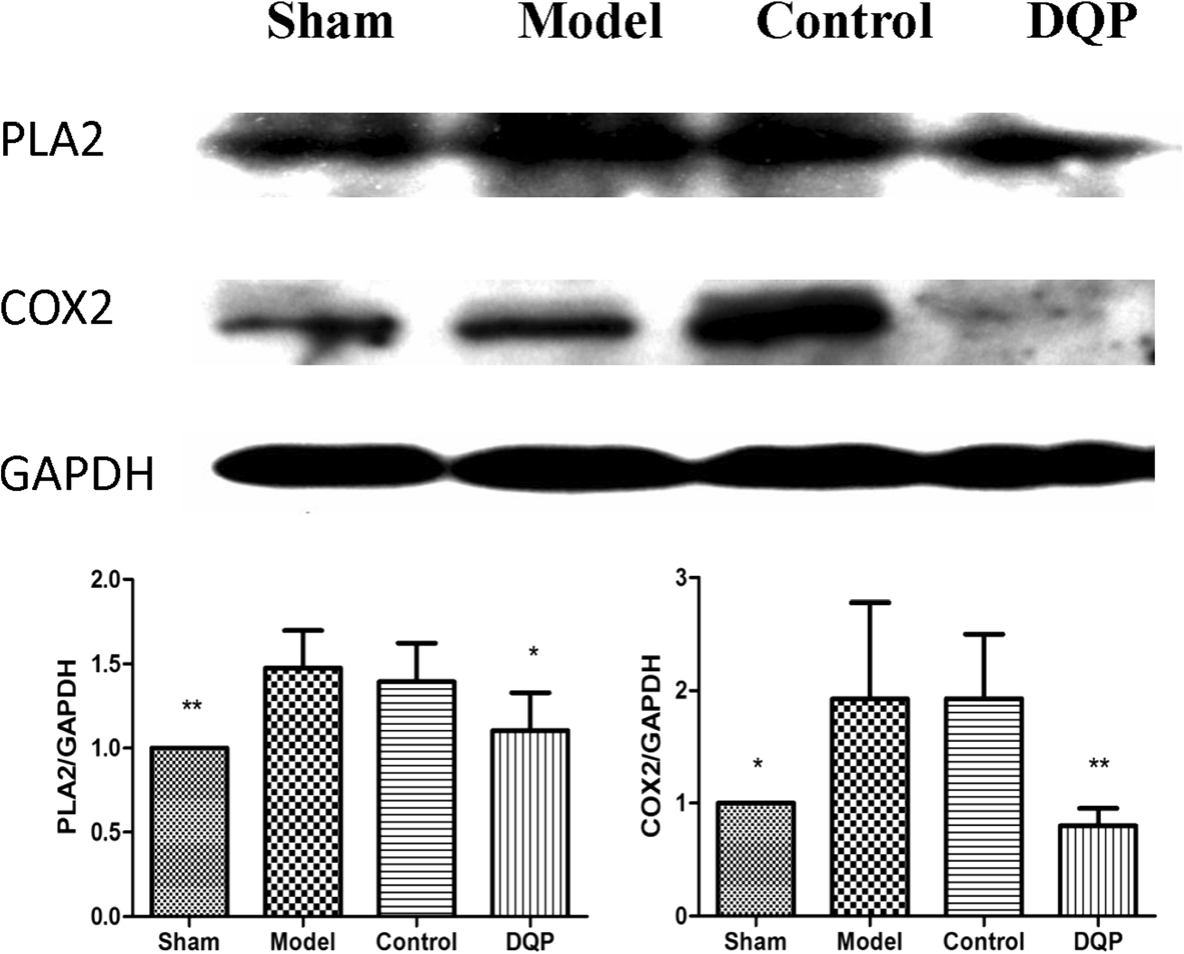
Effects of DQP on the myocardial concentrations of PLA2 and COX2. Western blot results showed that compared with the sham group, expressions of PLA2 and COX-2 in the model group were up-regulated. Expressions of PLA2 and COX-2 in DQP group were down-regulated significantly, compared with those in the model group. Control drug had no significant effect on the expression of PLA2 or COX-2. *P < 0.05, **P < 0.01. Protein expression levels in the model group (n = 6) were used as reference to calculate *P* values

We then investigated changes of proteins in PLA2-COX-2 mediated inflammation pathway. Compared with the sham group (1.00 ± 0.00, 1.00 ± 0.00), expressions of PLA2 and COX-2 in the model group were up-regulated by 47.5 % (1.48 ± 0.225, *P* = 0.002) and 92.54 % (1.93 ± 0.856, *P* = 0.0196), respectively. DQP was shown to inhibit the activation of this pathway. Expressions of PLA2 and COX-2 in DQP group were down-regulated by 25.11 % (1.10 ± 0.225, *P* = 0.012) and 58.50 % (0.799 ± 0.156, *P* = 0.0039), compared with those in the model group. Control drug had no significant effect on the expression of PLA2 or COX-2 (Fig. 3).

### Effects of DQP on PPARα signaling pathway

**Fig. 4:**
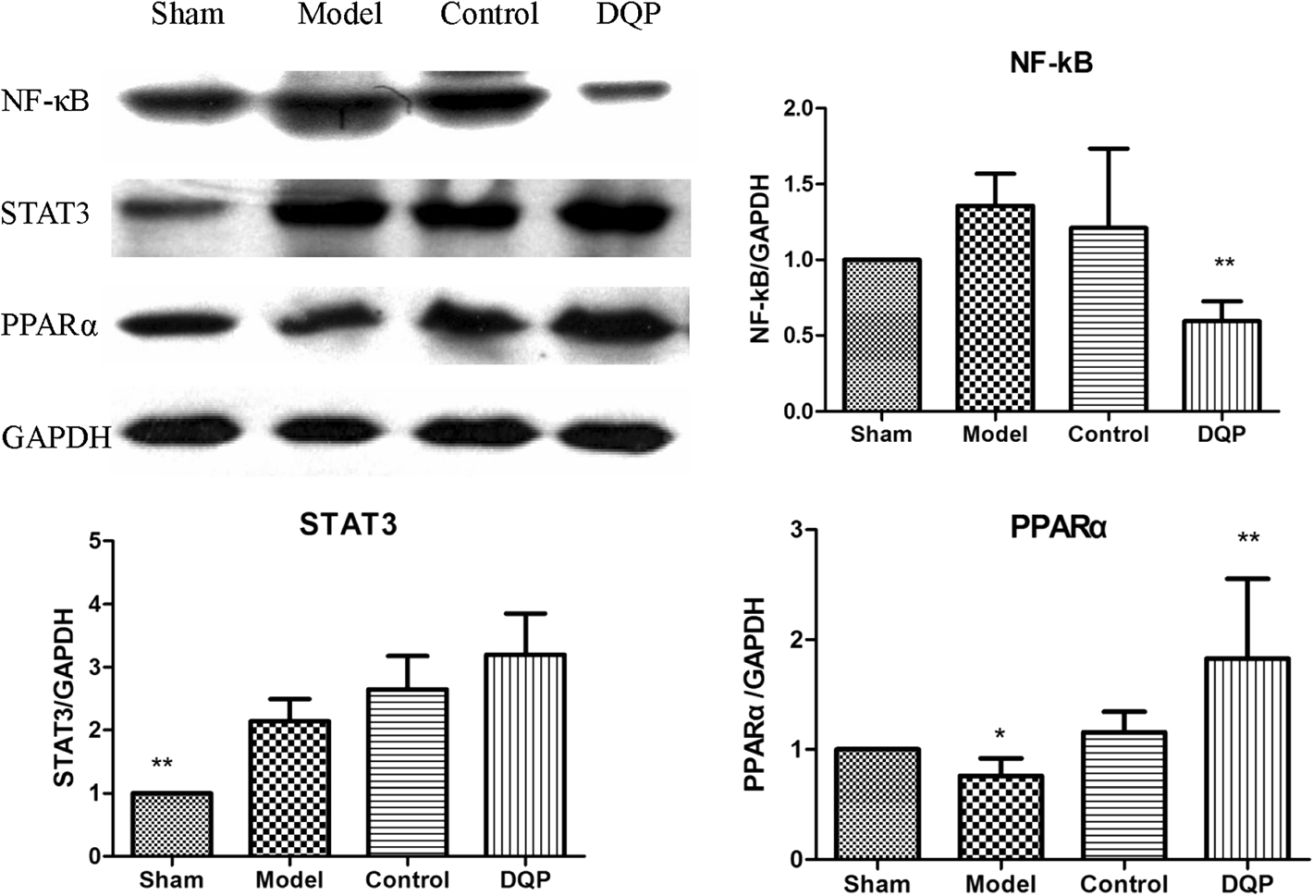
Effects of DQP on the levels of NF-κB, STAT3 and PPARα. Western blot showed that compared with the sham group, expressions of NF-κB and STAT3 in the model group were up-regulated (*P* = 0.074, P < 0.01). Expression of PPARα was down-regulated (*P* = 0.031) in the model group compared with that in the sham group. DQP could inhibit NF-κB level but showed no effect on STAT3 expression. Pravastatin had no effect on expression of STAT3 either. Compared with the model group, PPARα level increased in DQP group. *P < 0.05, **P < 0.01. Levels in the model group (n = 6) were used as reference to calculate *P* values

NF-κB and STAT3 are signal transduction factors that are important in promoting ventricular hypertrophy. Compared with the sham group (1.00 ± 0.00, 1.00 ± 0.00), expressions of NF-κB and STAT3 in the model group were up-regulated by 35.75 % (1.358 ± 0.212, *P* = 0.074) and 113.9 % (2.14 ± 0.354, *P* < 0.01), respectively. Expression of PPARα, the critical regulator of lipid metabolism, was down-regulated by 24.25 % (0.76 ± 0.162, *P* = 0.031) in the model group compared with that in the sham group. After treatment with DQP, NF-κB was inhibited and PPARα was activated. Expression of NF-κB was down-regulated by 56.2 % (0.59 ± 0.131, *P* = 0.0004) in DQP group compared with that in the model group, which suggested that the anti-inflammatory effect of DQP may be mediated by inhibition NF-κB. Compared with the model group, PPARα level increased by 141.31 % (1.83 ± 0.729, *P < 0.001*) in DQP group, demonstrating that DQP is a potent agonist of PPARα (Fig. 4). DQP showed no effect on STAT3 expression.

## Discussion

Traditional Chinese medicine has been widely used in the prevention and treatment of CHD for thousands of years [33]. DQP is one of the most commonly prescribed formulas and has been shown to be effective in treating CHD [34]. In this study, we investigated the pharmacological mechanisms of DQP in a rat model of CHD induced by ligation of coronary artery. Our major findings are as follows: 1), DQP can effectively improve the cardiac functions and regulate plasma lipids levels in rats with CHD. 2), DQP promotes lipid oxidation in cardiomyocytes by up-regulating expressions of molecules involved in uptake and transportation of lipids, which include ApoA-I, CD36 and CPT-1A. 3), the anti-inflammatory effect of DQP may be mediated by its inhibition on PLA2-COX-2 pathway. DQP down-regulates NF-κB expression, without affecting STAT3 expression. 4), DQP has the similar effect with that of PPARα agonist, and activation of PPARα might be the major mechanism by which DQP regulates lipid metabolism and inhibits inflammation.

It is reported that dyslipidemia is a high risk factor for CHD [35]. Cholesterol, triglycerides, LDL and HDL are the major components of plasma lipids that play critical roles in the development of CHD. Total blood cholesterol and LDL increase CHD risk in an independent manner [7, 36]. Elevated level of TG affects the process of reverse cholesterol transportation by HDL [37, 38]. It’s reported that an increase of 23 mg/dl in TG content will lead to 20 % increased risk of developing CHD [39]. Contrary to the effects of TG and LDL, HDL is considered to be a protective factor against development of CHD. High level of HDL is related with decreased risk of CHD [40]. HDL and its major component, ApoA-I, can promote reversal cholesterol transport and regress formation of atherosclerotic plaques [41, 42]. HDL can also inhibit inflammation by down-regulating expression of vascular cell adhesion molecule-1 [43]. In this study, we found that plasma levels of TG, LDL and VLDL increased, while levels of ApoA-I and HDL decreased in model group. Furthermore, level of lipoprotein lipase (LPL), the key enzyme in regulating lipid and lipoprotein metabolism, also decreased in the model group. Treatment with DQP could effectively regulate disorders in lipid metabolism, as was evidenced by decreased plasma levels of TG, LDL and increased HDL level in DQP group compared with the model group. However, DQP didn’t show effect on expression of LPL. Positive control drug showed similar effect on lipids levels as that of DQP. However, positive control drug failed to improve cardiac functions, indicating that DQP has advantage over control drug in the treatment of cardiovascular disease.

The effect of DQP on lipid metabolism pathway was then examined. Fatty acid translocase/cluster of differentiation protein (FAT/CD36) plays an important role in lipid metabolism by transporting long chain fatty acid into mitochondria [44–47]. Overexpression of FAT/CD36 promotes oxidation of fatty acid and lipoproteins [48]. Plasma fatty acids are translocated into cytoplasm of myocardial cells by binding with fatty acid binding protein (FABP) on the membrane, and are further transferred into mitochondria catalyzed by the enzyme CPT-I. Oxidation of fatty acids in mitochondria provides the majority of energy for heart contraction and CPT-I is the critical enzyme in oxidation process [49]. CPT-1A is one of the most abundant subtypes of CPT-I. In this study, expressions of FABP, CD36 and CPT-1A in the heart tissues of model rats were significantly reduced, which suggested that uptake and transportation of lipids were deregulated under myocardial ischemic conditions. The disorder of lipid transportation will lead to build-up of lipids in the plasma as well as shortage of energy supplies. After treatment with DQP, CD36 and CPT-1A levels were considerable up-regulated, compared with those in the model group, demonstrating that DQP has the effect of promoting lipid uptake by cardiac cells.

In addition to disorders of lipid metabolism, arachidonic acids (AA)-mediated inflammation is also an important pathological mechanism of cardiovascular diseases [25]. We further explored the effect of DQP on inflammation pathway. Under normal conditions, most of theAA exist in membranes as the components of phospholipids. When cell is under ischemic or inflammatory stimuli, phospholipid lipase A2 (PLA2) will catalytically hydrolyse phospholipids and release AA into cytoplasm. AA are subsequently converted to different metabolites by a series of enzymes. COXs are a group of enzymes that convert AA into prostaglandins (PGs) and thromboxanes (TXs), which are the major best-known products of AA. PGs and TXs mediate myocardial damage, apoptosis, fibrosis and progression of CHD [50]. Therefore, inhibiting activities of COXs has become a new strategy for the management of CHD [18]. Studies have shown that selective COX-2 inhibitors can inhibit vascular inflammation, reduce monocyte infiltration, increase production of NO, attenuate atherosclerosis and stabilize plaques, thereby reducing incidence of cardiovascular events [51]. COX-2 inhibitors can also improve cardiac function under myocardial infarction conditions [18]. In this study, we found that AA-mediated inflammation pathway was activated in the model group, as both PLA2 and COX-2 were over-expressed in ischemic heart tissues. DQP treatment significantly inhibited expressions of PLA2 and COX-2, indicating that DQP has both lipid-lowing and anti-inflammatory effects.

DQP also exerts its cardioprotective effects by acting on PPARα. There are three subtypes of PPAR: α, β(δ) and γ [52]. PPARα is mainly expressed in metabolically active tissues, such as heart, liver, kidney, etc. As a important regulator of metabolism, PPARα has a central coordinating role in the regulation of fatty acid oxidation, lipoprotein metabolism, inflammation and vascular responses [53–55]. It was reported that under myocardial ischemic circumstances, mRNA expression of PPARα was reduced and FFA uptake was decreased, aggravating cardiac ischemic damage [56]. All these evidences support a linkage between PPARα and cardiovascular diseases. PPARα also exerts anti-inflammatory effect by inhibiting NF-κB mediated pathway and down-regulating secretion of pro-inflammatory factors, such as IL-6, TNF-α and COX-2 [57]. Our study showed that in ischemic heart tissue, expression of PPARα was down-regulated and expressions of COX-2 and NF-κB were up-regulated, indicating that inflammation pathway was activated under ischemic conditions. DQP can effectively inhibit inflammation by reducing expressions of NF-κB and COX-2. More importantly, PPARα expression was significantly up-regulated in DQP-treated group compared with the model group, demonstrating that DQP is a potential effective PPARα agonist in the treatment of CHD.

## Conclusions

**Fig. 5:**
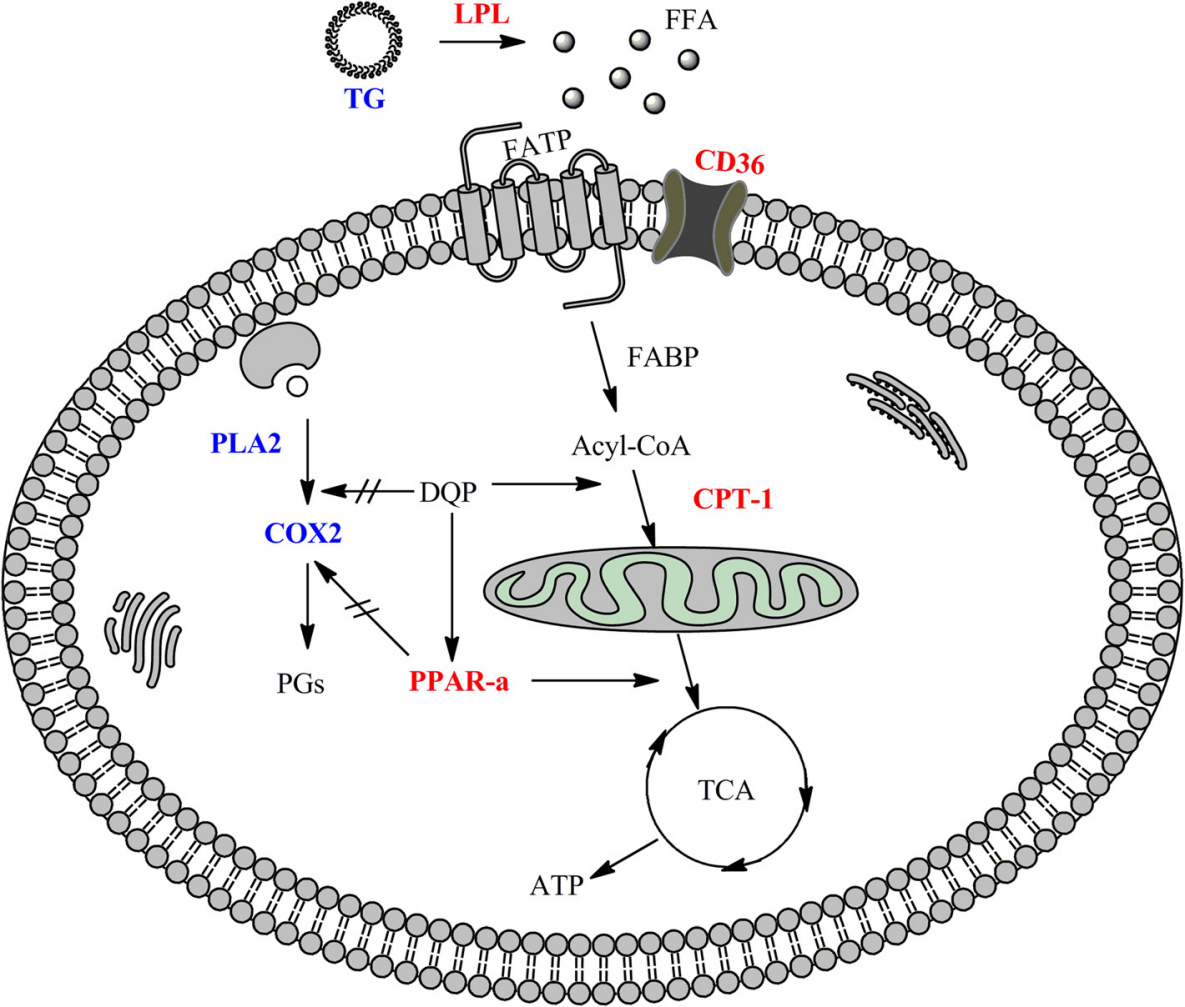
Potential mechanism of DQP efficacy on CHD rats. DQP has regulatory effect on lipid uptake-transport-metabolism pathway in myocardial cells of rats with heart failure. Moreover, DQP can up-regulate expression of PPARα significantly. The anti-inflammatory effect of DQP is probably achieved by inhibiting NF-κB in PLA2-COXs mediated inflammation pathway

DQP has regulatory effect on lipid uptake-transport-metabolism pathway in myocardial cells of rats with heart failure, and the effect is achieved mainly by activating ApoA-I-CD36-CPT-1A molecules. The anti-inflammatory effect of DQP is probably achieved by inhibiting NF-κB in PLA2-COXs mediated inflammation pathway (Fig. 5). Interestingly, DQP can up-regulate expression of PPARα significantly. This study provides experimental evidence for the clinical application of DQP in the treatment of CHD.

### Availability of data and materials

We have presented all our main data in the form of figures and tables. The datasets supporting the conclusions of this article are included within the article (and its additional file(s)).”

## References

[1] DiNicolantonio JJ, Lucan SC, O’Keefe JH. The evidence for saturated fat and for sugar related to coronary heart disease. Prog Cardiovasc Dis. 2015. 10.1016/j.pcad.2015.11.006 PMC4856550 26586275

[2] Wu T, Sun QF, Yang PS, Feng W, Liu XL (2010). [Levels of inflammation cytokines in patients with coronary heart disease and periodontal disease]. Zhonghua Kou Qiang Yi Xue Za Zhi.

[3] Ma Y, Ockene IS, Rosal MC, Merriam PA, Ockene JK, Gandhi PJ (2010). Randomized trial of a pharmacist-delivered intervention for improving lipid-lowering medication adherence among patients with coronary heart disease. Cholesterol.

[4] Rosen VM, Taylor DC, Parekh H, Pandya A, Thompson D, Kuznik A, Waters DD, Drummond M, Weinstein MC (2010). Cost effectiveness of intensive lipid-lowering treatment for patients with congestive heart failure and coronary heart disease in the US. Pharmacoeconomics.

[5] Bittner V, Deng L, Rosenson RS, Taylor B, Glasser SP, Kent ST, Farkouh ME, Muntner P (2015). Trends in the use of nonstatin lipid-lowering therapy among patients with coronary heart disease: a retrospective cohort study in the medicare population 2007 to 2011. J Am Coll Cardiol.

[6] Dargel R (1989). The lipid infiltration theory of atherosclerosis. Z Med Lab Diagn.

[7] Graham I, Cooney MT, Bradley D, Dudina A, Reiner Z (2012). Dyslipidemias in the prevention of cardiovascular disease: risks and causality. Curr Cardiol Rep.

[8] Barter P (2004). HDL: a recipe for longevity. Atheroscler Suppl.

[9] Mulay V, Wood P, Rentero C, Enrich C, Grewal T (2012). Signal transduction pathways provide opportunities to enhance HDL and apoAI-dependent reverse cholesterol transport. Curr Pharm Biotechnol.

[10] Tuunanen H, Ukkonen H, Knuuti J (2008). Myocardial fatty acid metabolism and cardiac performance in heart failure. Curr Cardiol Rep.

[11] Cook GA, Edwards TL, Jansen MS, Bahouth SW, Wilcox HG, Park EA (2001). Differential regulation of carnitine palmitoyltransferase-I gene isoforms (CPT-I alpha and CPT-I beta) in the rat heart. J Mol Cell Cardiol.

[12] Mengi SA, Dhalla NS (2004). Carnitine palmitoyltransferase-I, a new target for the treatment of heart failure: perspectives on a shift in myocardial metabolism as a therapeutic intervention. Am J Cardiovasc Drugs.

[13] Wang Y, Li C, Wang Q, Shi T, Wang J, Chen H, Wu Y, Han J, Guo S, Wang W (2015). Danqi Pill regulates lipid metabolism disorder induced by myocardial ischemia through FATP-CPTI pathway. BMC Complement Altern Med.

[14] Kantor PF, Lucien A, Kozak R, Lopaschuk GD (2000). The antianginal drug trimetazidine shifts cardiac energy metabolism from fatty acid oxidation to glucose oxidation by inhibiting mitochondrial long-chain 3-ketoacyl coenzyme A thiolase. Circ Res.

[15] Marchant DJ, Boyd JH, Lin DC, Granville DJ, Garmaroudi FS, McManus BM. Inflammation in myocardial diseases. Circ Res. 2012;110(1):126-44. 10.1161/CIRCRESAHA.111.243170 22223210

[16] Chandrasekharan NV, Simmons DL (2004). The cyclooxygenases. Genome Biol.

[17] Surh YJ, Chun KS, Cha HH, Han SS, Keum YS, Park KK, Lee SS (2001). Molecular mechanisms underlying chemopreventive activities of anti-inflammatory phytochemicals: down-regulation of COX-2 and iNOS through suppression of NF-kappa B activation. Mutat Res.

[18] LaPointe MC, Mendez M, Leung A, Tao ZY, Yang XP (2004). Inhibition of cyclooxygenase-2 improves cardiac function after myocardial infarction in the mouse. Am J Physiol-Heart C.

[19] Ray WA, Stein CM, Daugherty JR, Hall K, Arbogast PG, Griffin MR (2002). COX-2 selective non-steroidal anti-inflammatory drugs and risk of serious coronary heart disease. Lancet.

[20] Robinson E, Grieve DJ (2009). Significance of peroxisome proliferator-activated receptors in the cardiovascular system in health and disease. Pharmacol Ther.

[21] Nissen SE, Wolski K (2007). Effect of rosiglitazone on the risk of myocardial infarction and death from cardiovascular causes. N Engl J Med.

[22] Rakhshandehroo M, Knoch B, Muller M, Kersten S. Peroxisome proliferator-activated receptor alpha target genes. PPAR Res. 2010;2010. 10.1155/2010/612089 PMC2948931 20936127

[23] Bocher V, Chinetti G, Fruchart JC, Staels B (2002). [Role of the peroxisome proliferator-activated receptors (PPARS) in the regulation of lipids and inflammation control]. J Soc Biol.

[24] Wilson JL, Duan R, El-Marakby A, Alhashim A, Lee DL (2012). Peroxisome Proliferator Activated Receptor-alpha Agonist Slows the Progression of Hypertension, Attenuates Plasma Interleukin-6 Levels and Renal Inflammatory Markers in Angiotensin II Infused Mice. PPAR Res.

[25] Wang Y, Li C, Liu Z, Shi T, Wang Q, Li D, Wu Y, Han J, Guo S, Tang B (2014). DanQi Pill protects against heart failure through the arachidonic acid metabolism pathway by attenuating different cyclooxygenases and leukotrienes B4. BMC Complement Altern Med.

[26] Chen J, Wang F, Lee FS, Wang X, Xie M (2006). Separation and identification of water-soluble salvianolic acids from Salvia miltiorrhiza Bunge by high-speed counter-current chromatography and ESI-MS analysis. Talanta.

[27] Wan JB, Lai CM, Li SP, Lee MY, Kong LY, Wang YT (2006). Simultaneous determination of nine saponins from Panax notoginseng using HPLC and pressurized liquid extraction. J Pharm Biomed Anal.

[28] Ministry of Health of the People’s Republic of China Pharmacopoeia Committee (2010). China pharmaceutical standards for formulations and the drug prescription of the ministry of health of the People’s Republic. Chin Med Sci Technol Press.

[29] Chen S, Liu J, Liu X, Fu Y, Zhang M, Lin Q, Zhu J, Mai L, Shan Z, Yu X (2011). Panax notoginseng saponins inhibit ischemia-induced apoptosis by activating PI3K/Akt pathway in cardiomyocytes. J Ethnopharmacol.

[30] Tu EY, Zhou YG, Wang ZH, Liang QS, Yang GT (2009). Effects of tanshinone II A on the myocardial hypertrophy signal transduction system protein kinase B in rats. Chin J Integr Med.

[31] Wang Y, Li C, Ouyang YL, Chuo WJ, Yu JD, Han J, Chen JX, Wang W (2012). The study on therapy effect of Danqi pill to renin-angiotensin-aldosterone system (RAAS) and lipid metabolism disorder in coronary heart disease. Afr J Pharm Pharmaco.

[32] Wang Y, Li C, Wang QY, Shi TJ, Wang J, Chen H, Wu Y, Han J, Guo SZ, Wang YY, et al. Danqi Pill regulates lipid metabolism disorder induced by myocardial ischemia through FATP-CPTI pathway. Bmc Complem Altern M. 2015;15. 10.1186/s12906-015-0548-0 PMC4355010 25885422

[33] Shang QH, Liu ZL, Chen KJ, Xu H, Liu JP (2012). A systematic review of xuezhikang, an extract from red yeast rice, for coronary heart disease complicated by dyslipidemia. Evid-Based Compl Alt.

[34] Gao ZY, Xu H, Shi DZ, Wen C, Liu BY (2012). Analysis on outcome of 5284 patients with coronary artery disease: The role of integrative medicine. J Ethnopharmacol.

[35] Rana JS, Visser ME, Arsenault BJ, Despres JP, Stroes ES, Kastelein JJ, Wareham NJ, Boekholdt SM, Khaw KT (2010). Metabolic dyslipidemia and risk of future coronary heart disease in apparently healthy men and women: the EPIC-Norfolk prospective population study. Int J Cardiol.

[36] Majeed F, Miller M (2008). Low high-density lipoprotein cholesterol: an important consideration in coronary heart disease risk assessment. Curr Opin Endocrinol Diabetes Obes.

[37] Greene DJ, Skeggs JW, Morton RE (2001). Elevated triglyceride content diminishes the capacity of high density lipoprotein to deliver cholesteryl esters via the scavenger receptor class B type I (SR-BI). J Biol Chem.

[38] Skeggs JW, Morton RE (2002). LDL and HDL enriched in triglyceride promote abnormal cholesterol transport. J Lipid Res.

[39] Carey VJ, Bishop L, Laranjo N, Harshfield BJ, Kwiat C, Sacks FM (2010). Contribution of high plasma triglycerides and low high-density lipoprotein cholesterol to residual risk of coronary heart disease after establishment of low-density lipoprotein cholesterol control. Am J Cardiol.

[40] Cui Y, Watson DJ, Girman CJ, Shapiro DR, Gotto AM, Hiserote P, Clearfield MB (2009). Effects of increasing high-density lipoprotein cholesterol and decreasing low-density lipoprotein cholesterol on the incidence of first acute coronary events (from the Air Force/Texas Coronary Atherosclerosis Prevention Study). Am J Cardiol.

[41] Ibanez B, Vilahur G, Cimmino G, Speidl WS, Pinero A, Choi BG, Zafar MU, Santos-Gallego CG, Krause B, Badimon L (2008). Rapid change in plaque size, composition, and molecular footprint after recombinant apolipoprotein A-I Milano (ETC-216) administration: magnetic resonance imaging study in an experimental model of atherosclerosis. J Am Coll Cardiol.

[42] Nicholls SJ, Gordon A, Johannson J, Ballantyne CM, Barter PJ, Brewer HB, Kastelein JJP, Wong NC, Borgman MRN, Nissen SE (2012). ApoA-I induction as a potential cardioprotective strategy: rationale for the SUSTAIN and ASSURE studies. Cardiovasc Drug Ther.

[43] Cockerill GW, Rye KA, Gamble JR, Vadas MA, Barter PJ (1995). High-density-lipoproteins inhibit cytokine-induced expression of endothelial-cell adhesion molecules. Arterioscl Throm Vas.

[44] Berk PD (2008). Regulatable fatty acid transport mechanisms are central to the pathophysiology of obesity, fatty liver, and metabolic syndrome. Hepatology.

[45] Luiken JJFP, Koonen DPY, Willems J, Zorzano A, Becker C, Fischer Y, Tandon NN, van der Vusse GJ, Bonen A, Glatz JFC (2002). Insulin stimulates long-chain fatty acid utilization by rat cardiac myocytes through cellular redistribution of FAT/CD36. Diabetes.

[46] Tanaka T, Nakata T, Oka T, Ogawa T, Okamoto F, Kusaka Y, Sohmiya K, Shimamoto K, Itakura K (2001). Defect in human myocardial long-chain fatty acid uptake is caused by FAT/CD36 mutations. J Lipid Res.

[47] Smith BK, Jain SS, Rimbaud S, Dam A, Quadrilatero J, Ventura-Clapier R, Bonen A, Holloway GP (2011). FAT/CD36 is located on the outer mitochondrial membrane, upstream of long-chain acyl-CoA synthetase, and regulates palmitate oxidation. Biochem J.

[48] He JH, Lee JH, Febbraio M, Xie W (2011). The emerging roles of fatty acid translocase/CD36 and the aryl hydrocarbon receptor in fatty liver disease. Exp Biol Med.

[49] Bruce CR, Hoy AJ, Turner N, Watt MJ, Allen TL, Carpenter K, Cooney GJ, Febbraio MA, Kraegen EW (2009). Overexpression of carnitine palmitoyltransferase-1 in skeletal muscle is sufficient to enhance fatty acid oxidation and improve high-fat diet-induced insulin resistance. Diabetes.

[50] Levick SP, Loch DC, Taylor SM, Janicki JS (2007). Arachidonic acid metabolism as a potential mediator of cardiac fibrosis associated with inflammation. J Immunol.

[51] Pitt B, Pepine C, Willerson JT (2002). Cyclooxygenase-2 inhibition and cardiovascular events. Circulation.

[52] Issemann I, Prince RA, Tugwood JD, Green S (1993). The peroxisome proliferator-activated receptor retinoid-x receptor heterodimer is activated by fatty-acids and fibrate hypolipemic drugs. J Mol Endocrinol.

[53] Auwerx J, Schoonjans K, Fruchart JC, Staels B (1996). Transcriptional control of triglyceride metabolism: fibrates and fatty acids change the expression of the LPL and apo C-III genes by activating the nuclear receptor PPAR. Atherosclerosis.

[54] Schoonjans K, Staels B, Auwerx J (1996). Role of the peroxisome proliferator-activated receptor (PPAR) in mediating the effects of fibrates and fatty acids on gene expression. J Lipid Res.

[55] Fruchart JC (2009). Peroxisome proliferator-activated receptor-alpha (PPAR alpha): at the crossroads of obesity, diabetes and cardiovascular disease. Atherosclerosis.

[56] Yue TL, Bao W, Jucker BM, Gu JL, Romanic AM, Brown PJ, Cui JQ, Thudium DT, Boyce R, Burns-Kurtis CL (2003). Activation of peroxisome proliferator-activated receptor-alpha protects the heart from ischemia/reperfusion injury. Circulation.

[57] Shonde A, Bennett A, Hawkey CJ (2007). Suppression of cyclooxygenase (COX)-2 and cellular proliferation in colonocytes by indornethacin: role of leukotriene (LT)B4, a (Ppar)alpha ligand. Gastroenterology.

